# Supplementary figures and images for: Forward genetics in C. elegans reveals genetic adaptations to polyunsaturated fatty acid deficiency
Source: eLife. 2025 Jul 8;13:RP104181. doi: 10.7554/eLife.104181 (PMC12237404; doi:10.7554/eLife.104181)

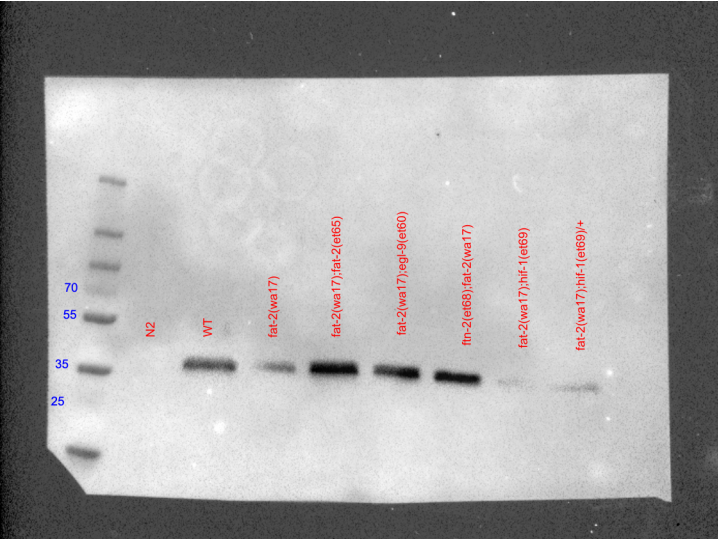

Supplement: Figure 6—source data 1. [file elife-104181-fig6-data1.zip › Figure 6_source data 1_PDFs annotated original blots/Fig_6D_HA labelled.pdf]

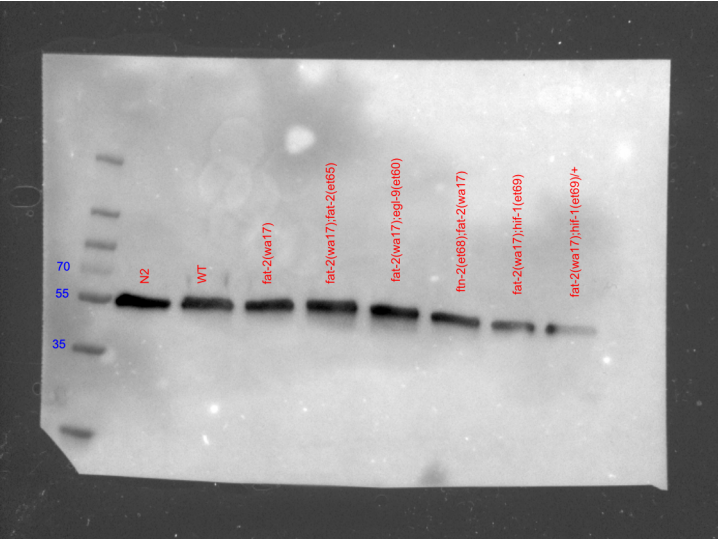

Supplement: Figure 6—source data 1. [file elife-104181-fig6-data1.zip › Figure 6_source data 1_PDFs annotated original blots/Fig_6D_tubulin labelled.pdf]

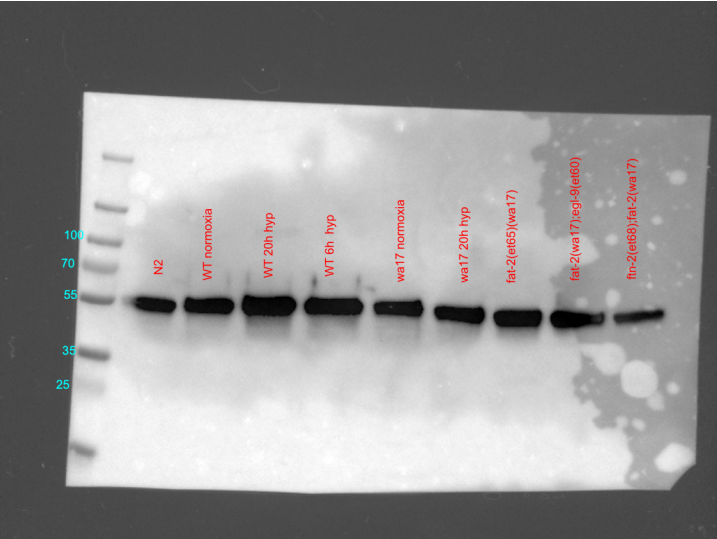

Supplement: Figure 6—source data 1. [file elife-104181-fig6-data1.zip › Figure 6_source data 1_PDFs annotated original blots/Fig_6A_tubulin labelled.pdf]

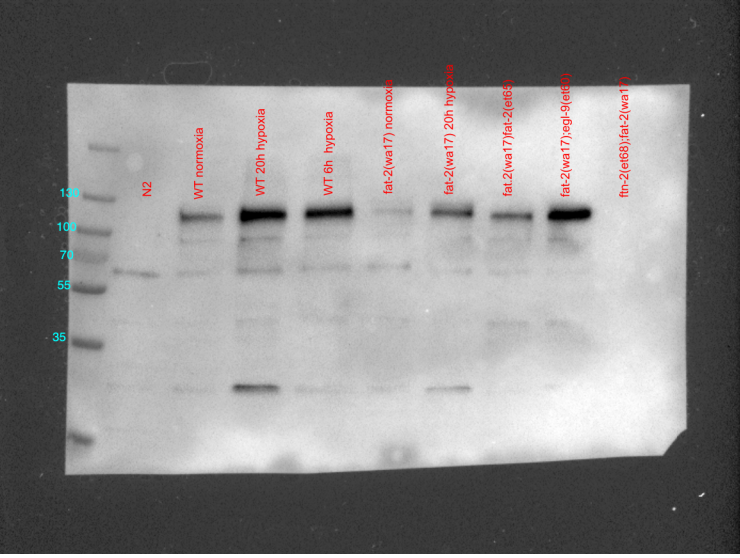

Supplement: Figure 6—source data 1. [file elife-104181-fig6-data1.zip › Figure 6_source data 1_PDFs annotated original blots/Fig_6A_FLAG labelled.pdf]

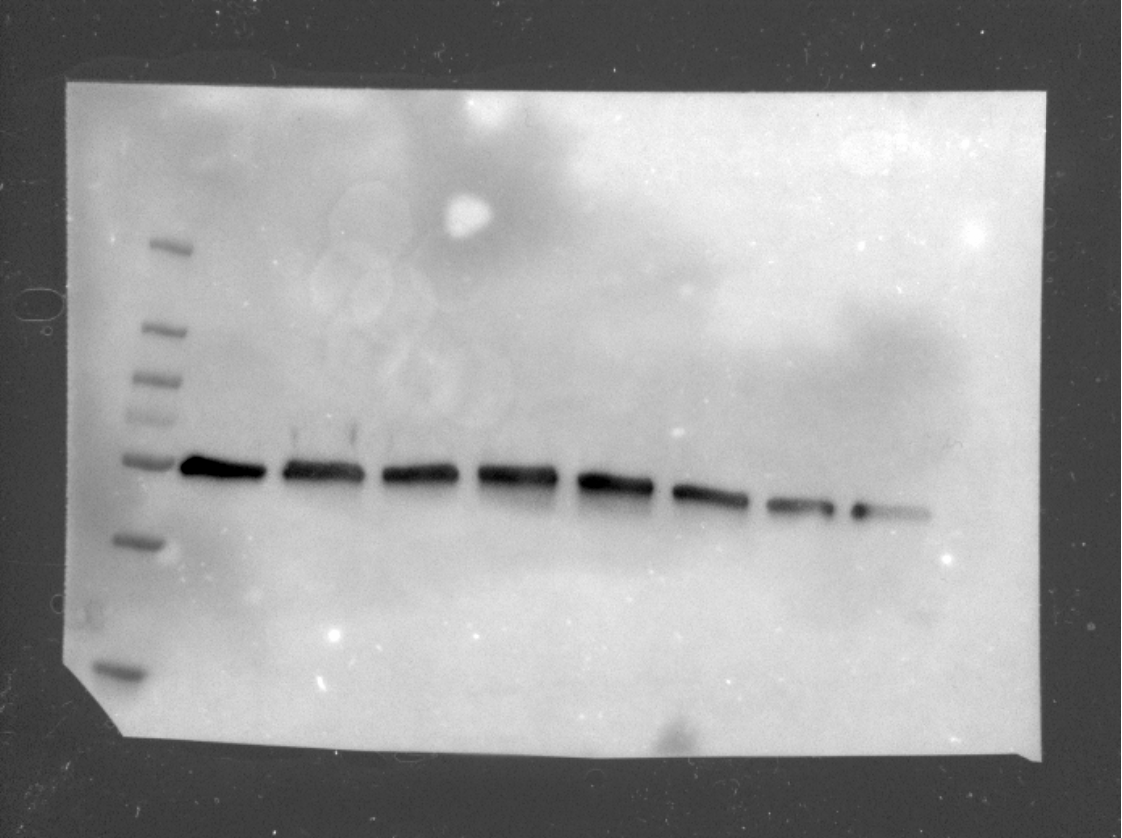

Supplement: Figure 6—source data 2. [file elife-104181-fig6-data2.zip › Figure 6_source data 2_Original files/Fig_6D_tubulin merged with ladder.tif]

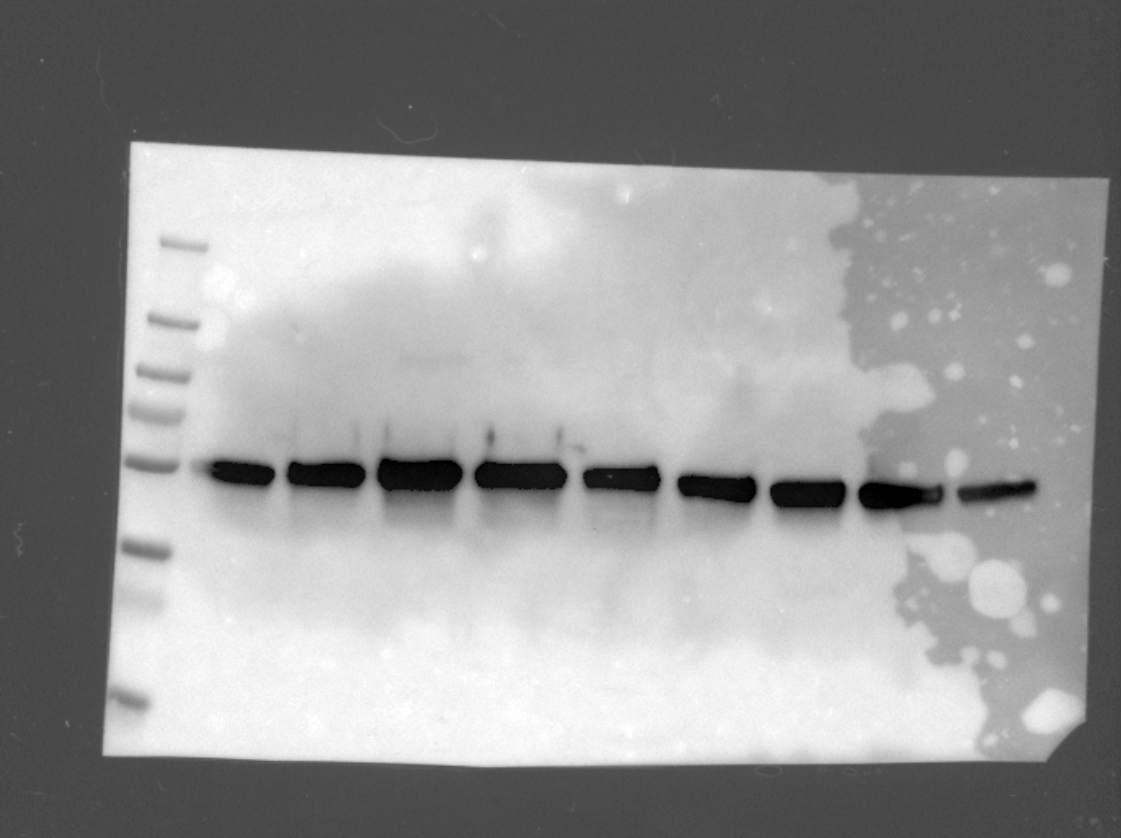

Supplement: Figure 6—source data 2. [file elife-104181-fig6-data2.zip › Figure 6_source data 2_Original files/Fig_6A_tubulin merged with ladder.tif]

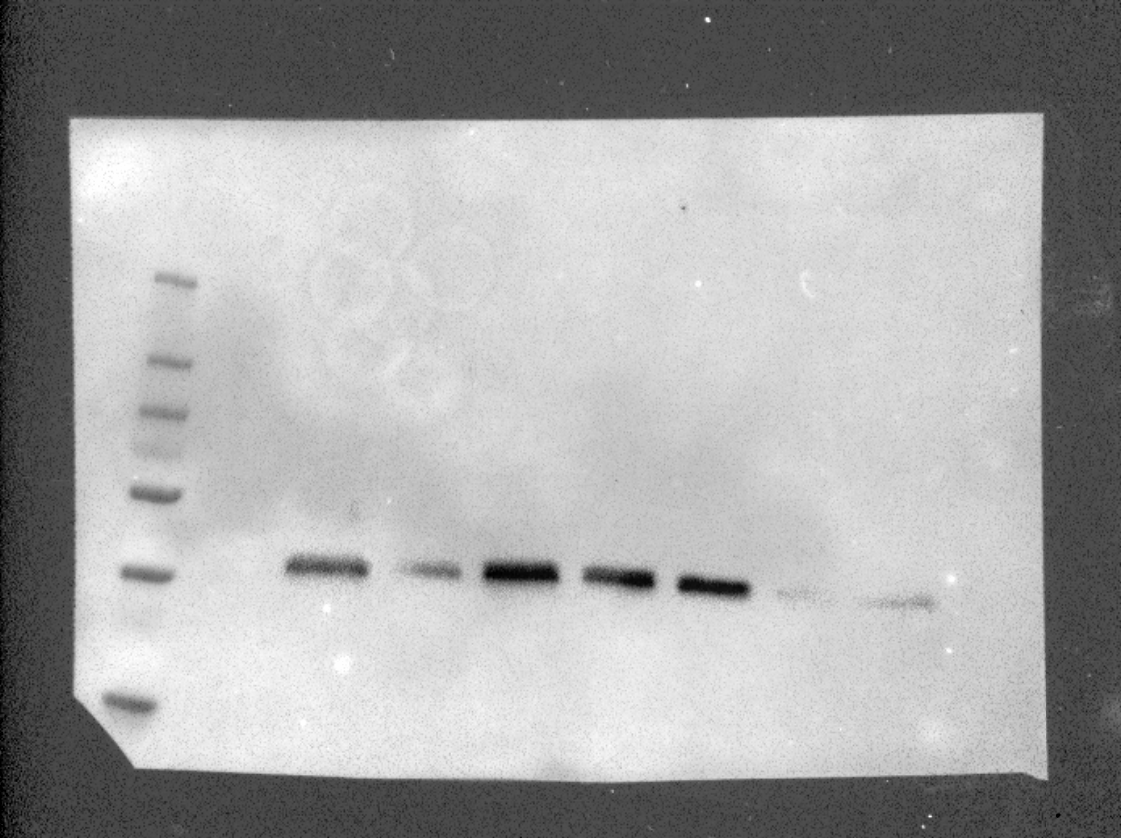

Supplement: Figure 6—source data 2. [file elife-104181-fig6-data2.zip › Figure 6_source data 2_Original files/Fig_6D_HA merged with ladder.tif]

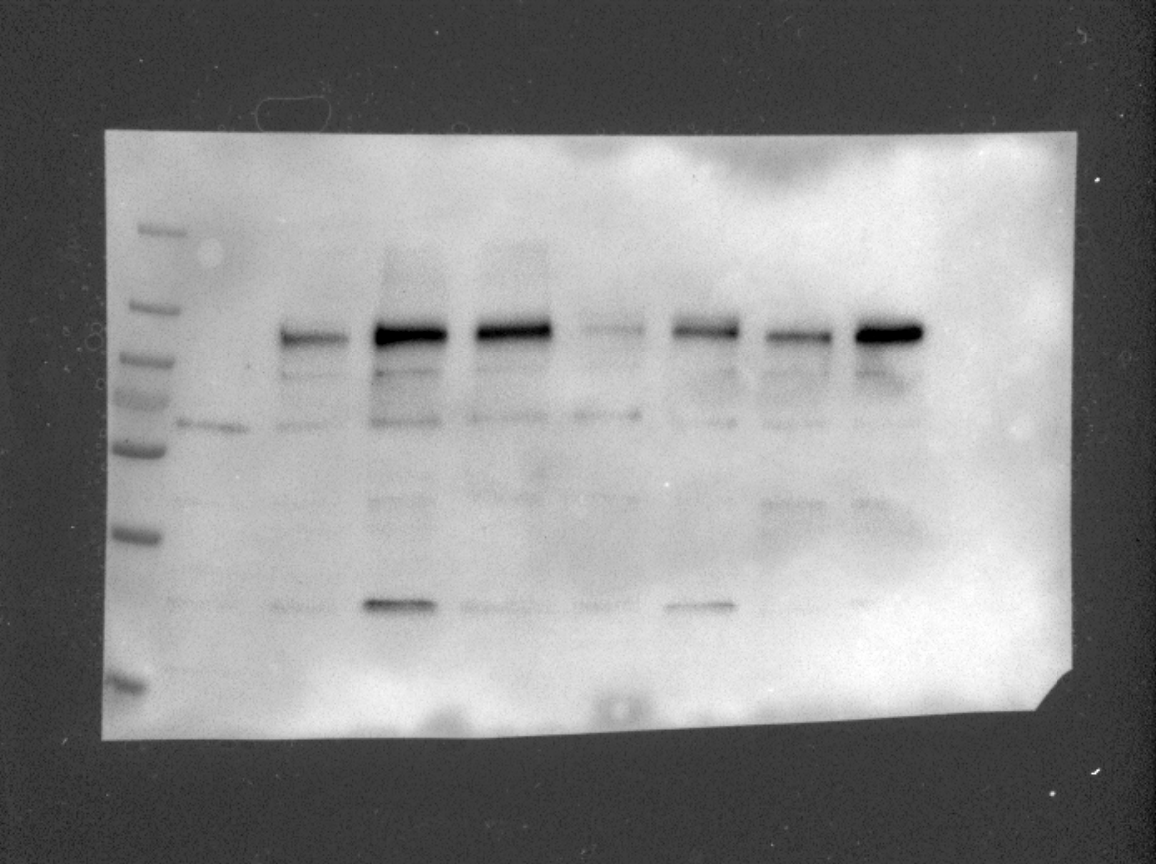

Supplement: Figure 6—source data 2. [file elife-104181-fig6-data2.zip › Figure 6_source data 2_Original files/Fig_6A_FLAG merged with ladder.tif]

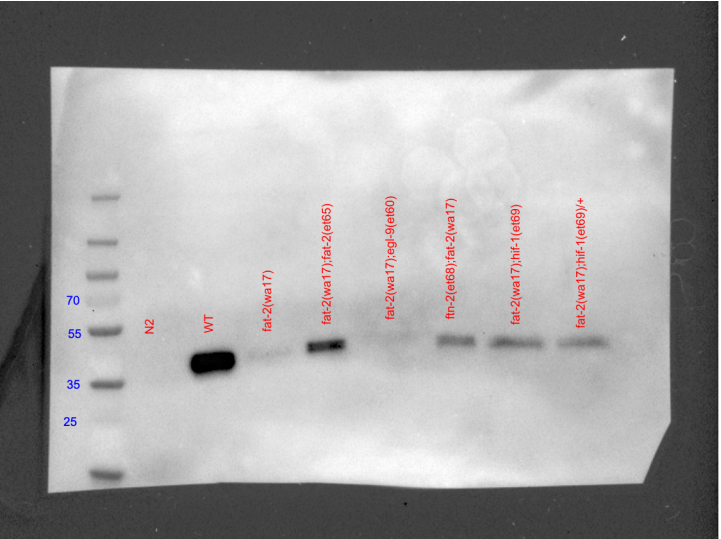

Supplement: Figure 6—figure supplement 1—source data 1. [file elife-104181-fig6-figsupp1-data1.zip › Figure 6_Fig_Suppl_1_source data 1_PDFs annotated original blots/Fig_6_Fig_Suppl_1C_HA labelled.pdf]

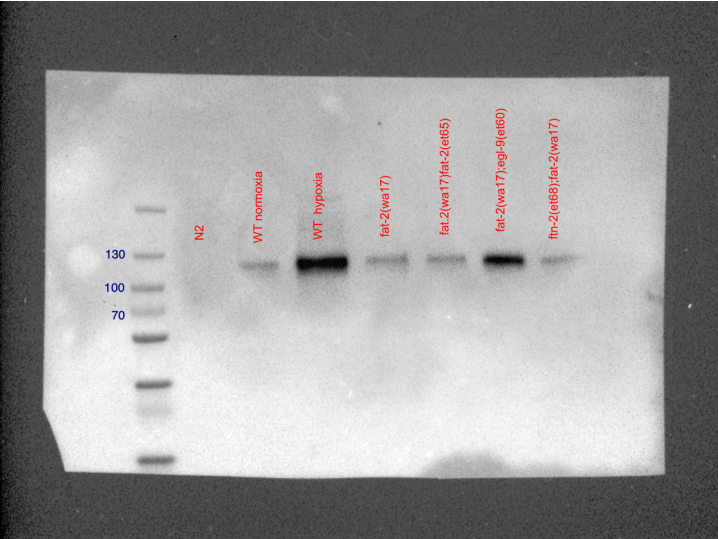

Supplement: Figure 6—figure supplement 1—source data 1. [file elife-104181-fig6-figsupp1-data1.zip › Figure 6_Fig_Suppl_1_source data 1_PDFs annotated original blots/Fig 6_Fig_Suppl 1A_FLAG labelled.pdf]

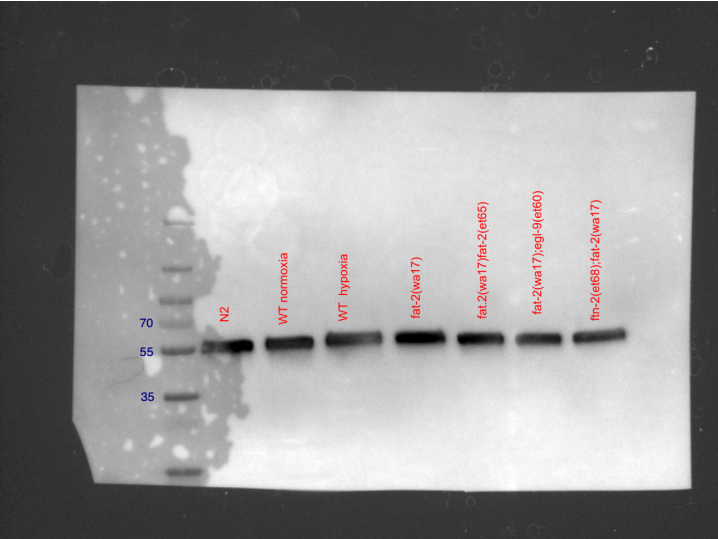

Supplement: Figure 6—figure supplement 1—source data 1. [file elife-104181-fig6-figsupp1-data1.zip › Figure 6_Fig_Suppl_1_source data 1_PDFs annotated original blots/Fig_6_Fig_Suppl_1A_tubulin labelled.pdf]

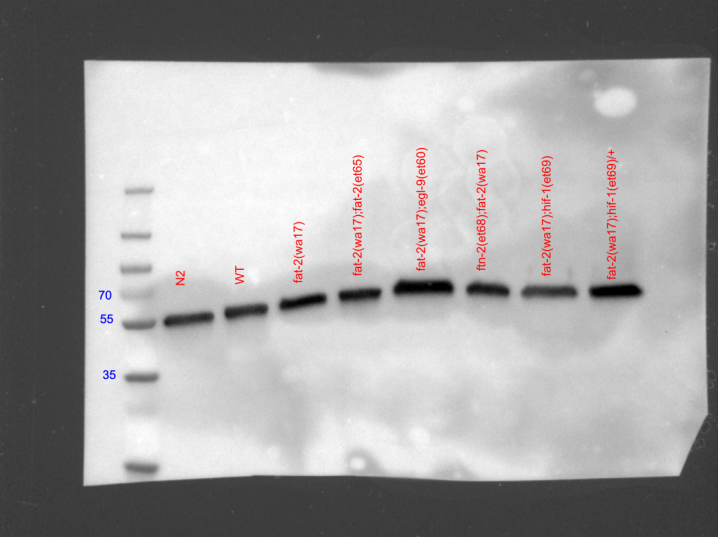

Supplement: Figure 6—figure supplement 1—source data 1. [file elife-104181-fig6-figsupp1-data1.zip › Figure 6_Fig_Suppl_1_source data 1_PDFs annotated original blots/Fig_6_Fig_Suppl_1C_tubulin labelled.pdf]

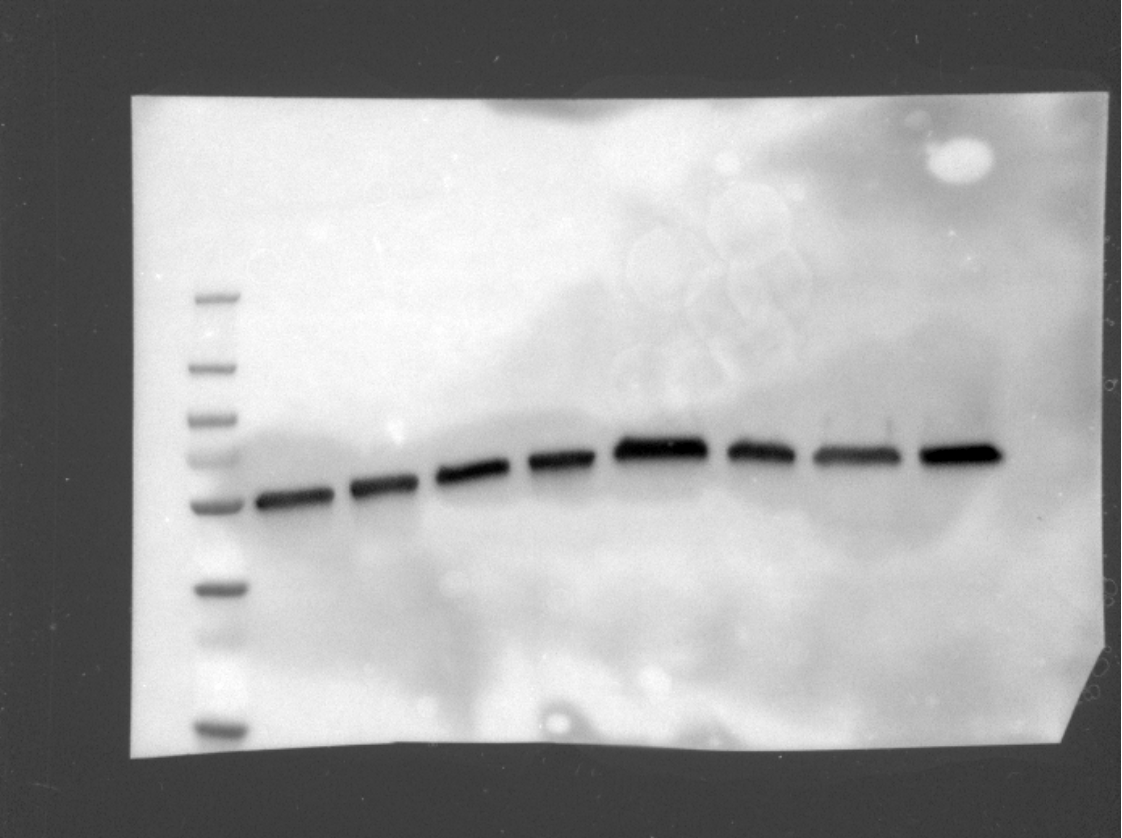

Supplement: Figure 6—figure supplement 1—source data 2. [file elife-104181-fig6-figsupp1-data2.zip › Figure 6_Fig_Suppl_1_source data 2_Original files/Fig_6_Fig_Suppl_1C_tubulin merged with ladder.tif]

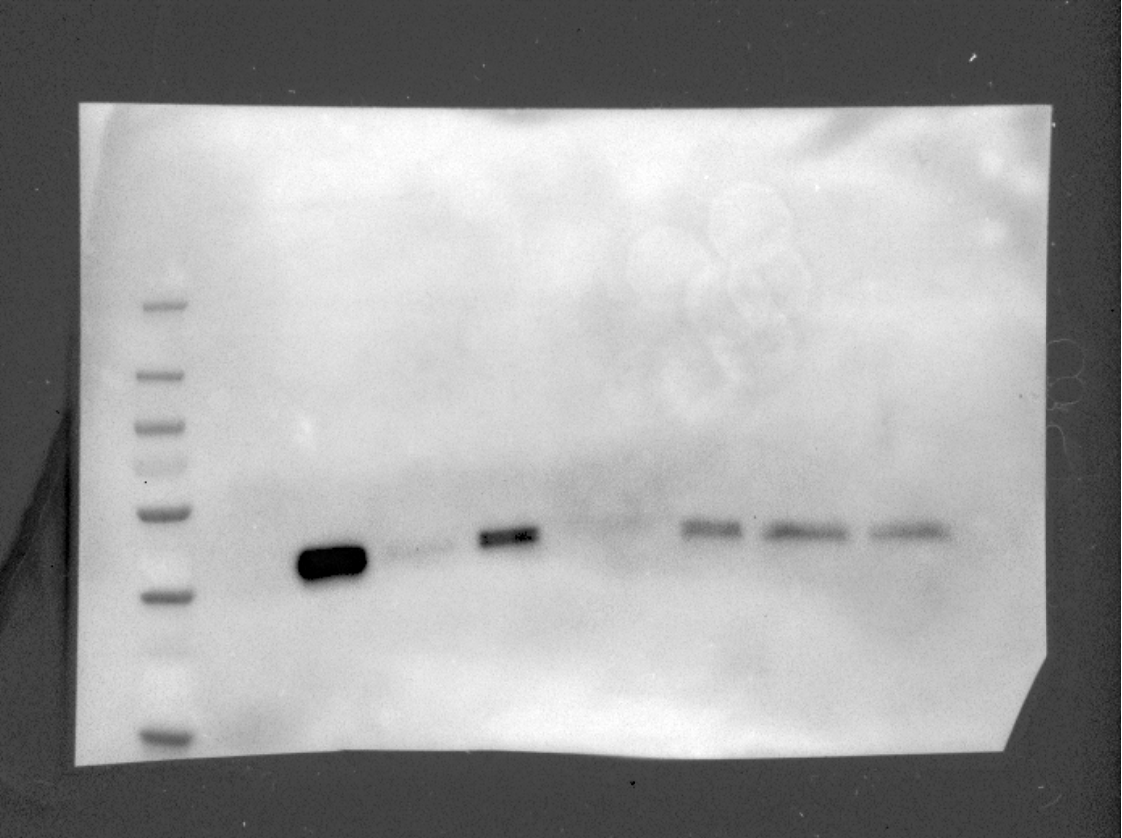

Supplement: Figure 6—figure supplement 1—source data 2. [file elife-104181-fig6-figsupp1-data2.zip › Figure 6_Fig_Suppl_1_source data 2_Original files/Fig_6_Fig_Suppl_1C_HA merged with ladder.tif]

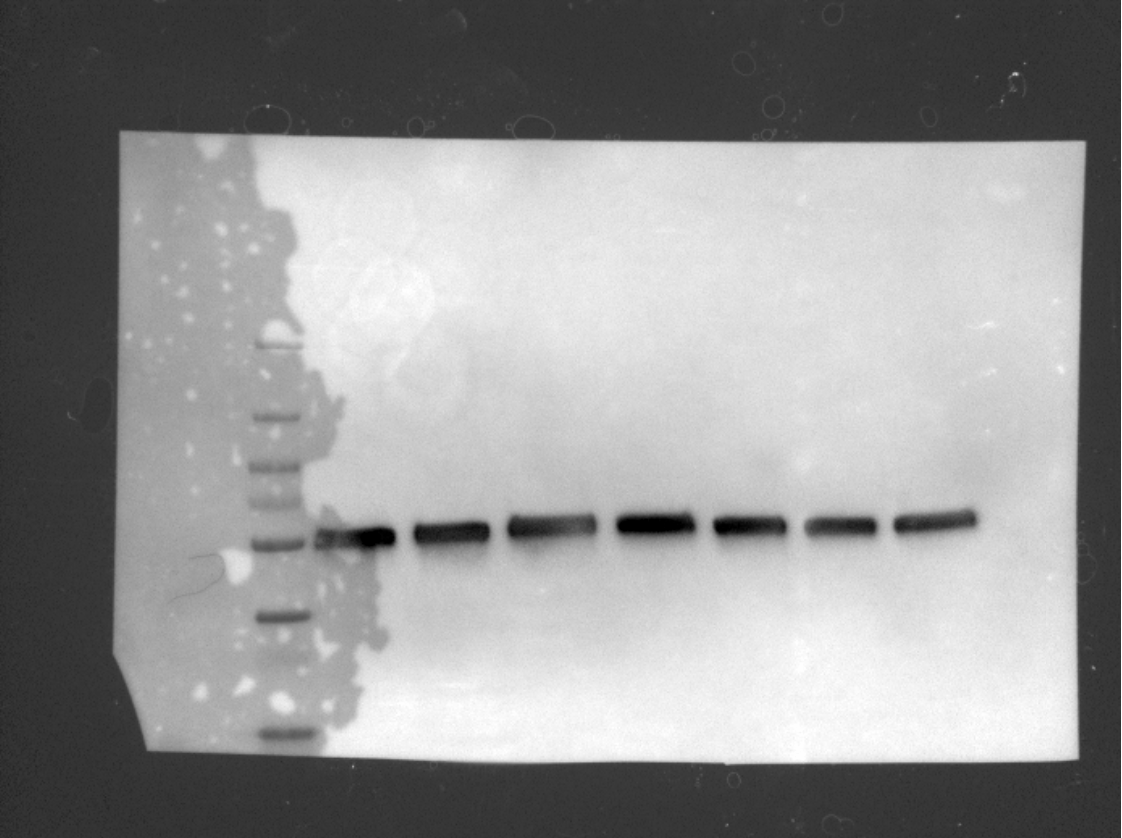

Supplement: Figure 6—figure supplement 1—source data 2. [file elife-104181-fig6-figsupp1-data2.zip › Figure 6_Fig_Suppl_1_source data 2_Original files/Fig_6_Fig_Suppl_1A_tubulin merged with ladder.tif]

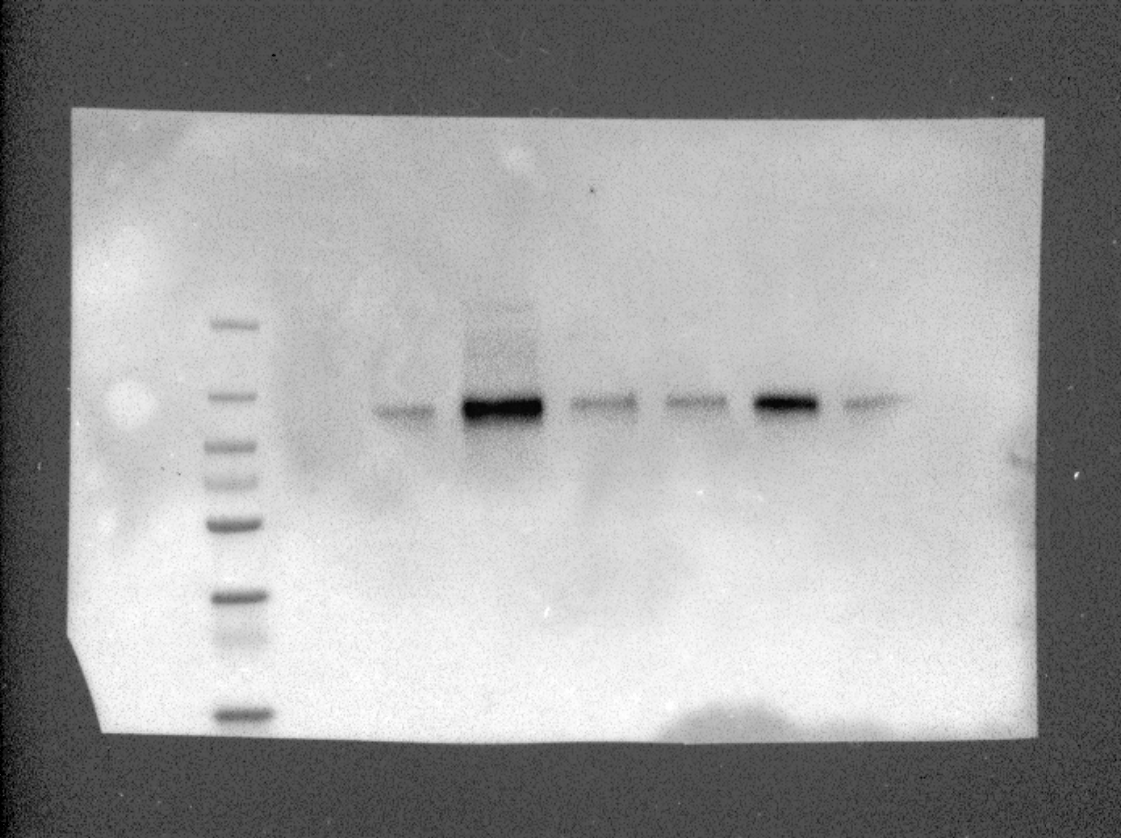

Supplement: Figure 6—figure supplement 1—source data 2. [file elife-104181-fig6-figsupp1-data2.zip › Figure 6_Fig_Suppl_1_source data 2_Original files/Fig_6_Fig_Suppl_1A_FLAG merged with ladder.tif]
